# Ultrabroadband High Photoresponsivity at Room Temperature Based on Quasi‐1D Pseudogap System (TaSe_4_)_2_I

**DOI:** 10.1002/advs.202302886

**Published:** 2023-12-08

**Authors:** Jialin Li, Qing Li, Junjian Mi, Zhuan Xu, Yu Xie, Wei Tang, Huanfeng Zhu, Linjun Li, Limin Tong

**Affiliations:** ^1^ State Key Laboratory of Modern Optical Instrumentation College of Optical Science and Engineering Zhejiang University Hangzhou 310027 China; ^2^ Hangzhou Institute for Advanced Study University of Chinese Academy of Sciences Hangzhou 310024 China; ^3^ Zhejiang Province Key Laboratory of Quantum Technology and Device Department of Physics Zhejiang University Hangzhou 310027 China; ^4^ Research Center for Humanoid Sensing Zhejiang Lab Hangzhou 311100 China; ^5^ Intelligent Optics and Photonics Research Center Jiaxing Research Institute Zhejiang University Jiaxing 314000 China; ^6^ Jiaxing Key Laboratory of Photonic Sensing and Intelligent Imaging Jiaxing Institute Zhejiang University Jiaxing 314000 China

**Keywords:** infrared photodetection, pseudogap, quasi 1D, (TaSe_4_)_2_I

## Abstract

Narrow bandgap materials have garnered significant attention within the field of broadband photodetection. However, the performance is impeded by diminished absorption near the bandgap, resulting in a rapid decline in photoresponsivity within the mid‐wave infrared (MWIR) and long‐wave infrared (LWIR) regions. Furthermore, they mostly worked in cryogenic temperature. Here, without the assistance of any complex structure and special environment, it is realized high responsivity covering ultra‐broadband wavelength range (Ultraviolet (UV) to LWIR) in a single quasi‐1D pseudogap (PG) system (TaSe_4_)_2_I nanoribbon, especially high responsivity (From 23.9 to 8.31 A W^−1^) within MWIR and LWIR region at room temperature (RT). Through direct probing the carrier relaxation process with broadband time‐resolved transient absorption spectrum measurement, the underlying mechanism of majorly photoconductive effect is revealed, which causes an increased spectral weight extended to PG region. This work paves the way for realizing high‐performance uncooled MWIR and LWIR detection by using quasi‐1D PG materials.

## Graphical abstract

A new type of photodetector based on PG system (TaSe_4_)_2_I is proposed, and high photo responsivity covering ultrabroadband wavelength range (UV to LWIR) including mechanism are revealed in proof‐of‐concept nanoribbon devices. The achieved photodetection performance is superior than most of other low‐dimensional materials. These results may pave a way for exploring advanced MWIR and LWIR photodetector at RT by using quasi‐1D PG materials.

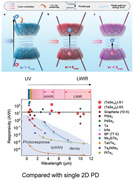

## 1 Introduction

High performance broadband photodetection ability, especially in the MWIR (3–5 µm) and LWIR (7–14 µm) range, is quite important for the applications such as thermal imaging, medical quarantine, and industrial monitoring. Narrow bandgap semiconductors such as HgCdTe,^[^ ^1^ ^]^ InGaAs,^[^ ^2^ ^]^ and InSb,^[^ ^3^ ^]^ are commercially used for MWIR and LWIR detection. However, they have complex preparation process and work at cryogenic temperature to reduce the dark current, which suffers from expensive cost. It is desirable to find novel and efficient photosensitive materials especially working in the middle and far‐infrared region at room temperature (RT). Over the past few years, a large amount of wide‐band photodetection devices based on narrow‐bandgap semiconductors^[^ ^4^ ^]^ or semimetal materials^[^ ^5^ ^]^ were explored. However, on one hand, due to the existence of near zero band gap, the devices usually have high dark current, restricting the improvement of photoresponsivity and detectivity, so it remains a challenge to achieve high‐performance photodetection in the MWIR and LWIR range at RT. On the other hand, orders of magnitude reduced absorption from visible to LWIR range leads to the low responsivity in middle and far IR.

For a traditional narrow bandgap semiconductor, the reduced absorption from short wavelength to long wavelength light has resulted from the reduced density of states when energy approaches the bandgap edge. To compensate the much lower absorption at longer wavelength, that is MWIR or LWIR, the mid gap states were intentionally introduced by generating defects^[^ ^6^ ^]^ and heterostructures combined with plasmonic quantum dots were explored.^[^ ^7^ ^]^ An extrinsic method to enhance the absorption in longer wavelength is to add external structures, such as cavity,^[^ ^8^ ^]^ optical antenna,^[^ ^9^ ^]^ plasmonic,^[^ ^10^ ^]^ metasurface^[^ ^11^ ^]^ or antireflection structure.^[^ ^12^ ^]^ Nevertheless, these methods are either uncontrollable or endure high fabrication cost, which hardly be used for stable, low cost MWIR or LWIR photoelectronic applications. Another approach to address this issue is the utilization of a combination of materials with varying bandgaps, wherein each bandgap material is responsible for absorbing light in its respective wavelength region. By exploiting bandgap engineering and quantum tunneling, the quantum cascade broadband IR photodetector (quantum cascade detector, QCD) was proposed and nowadays becomes the state‐of‐the‐art IR photodetector.^[^ ^13^ ^]^ However, due to the complex structure and the required advanced fabrication technique, the cost for producing and maintaining QCD is extremely high and the lifetime is still very limited. Therefore, a single material broadband IR photodetector with high photoresponse at all wavelength ranges is highly demanded.

Specifically, to improve the photoresponsivity at MWIR and LWIR detection in single material at room temperature, photogating and bolometric mechanism were proposed in previous studies. However, either the sample defects for photogating is not controllable, or the high‐temperature coefficient of resistance (TCR) for bolometric effect is needed.^[^ ^14^ ^]^ Recently, it has been demonstrated that 2D charge density wave (CDW) materials exhibit enhanced photo responsivity of ≈1 A W^−1^ due to their collective transport, thereby improving intrinsic photoconduction.^[^ ^15^ ^]^ The CDW gap is usually small and develops in a metallic ground state, which enables their high density of states near the gap edge, hence enabling high absorption for long wavelength light. Nevertheless, photodetectors based on 2D CDW materials are very unstable, due to the random distribution of CDW domains^[^ ^16^ ^]^ and in fact they worked as threshold detectors, which limits the achievement of higher performance.

**Figure 1:**
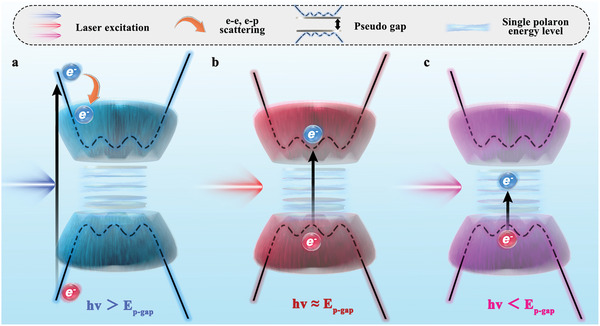
Energy band diagram and proposed concept of the photodetector based on PG system (TaSe_4_)_2_I at room temperature.

Naturally, the question arises as to how we can find a material with semiconducting ground state but with high density of states (DOS) near the bandgap edge available for broadband photoconduction. This question attracts our attention to the materials with PG. PG systems have previously attracted a great interest in condensed matter physics, especially the one related to the Mott superconducting transition of high‐T_c_ superconductor.^[^ ^17^ ^]^ Usually, such PG associates with the strong electron‐electron correlation state when the 2D Mott insulating system is doped to the boundary of “strange metal”^[^ ^18^ ^]^ or a 1D strongly corrected metal, such as Luttinger liquid state^[^ ^19^ ^]^ or bipolaron state.^[^ ^20^ ^]^ Since these systems have already large occupied DOS for photon excitation while the dark state is semiconducting instead, they are naturally the candidate materials for seeking equally high photoresponsivity from visible to MIR region. However, to date, the study for light modulating the materials with PG and its application in broadband photodetection is almost absent. In this work, we investigate the broadband photoresponse of 1D (TaSe_4_)_2_I, which has PG at room temperature. The investigation of PG in (TaSe_4_)_2_I was previously conducted^[^ ^21^ ^]^ and has been revisited in recent studies utilizing time‐resolved angle‐resolved photoelectron spectroscopy (trARPES).^[^ ^20^, ^22^ ^]^ The observed enhancement of spectral weight at Fermi energy (*E*_F_) under λ=780 nm photoexcitation suggests potential advantages for achieving high performance photodetection.^[^ ^22^ ^]^ Herein, we investigate the photoresponse of 1D (TaSe_4_)_2_I and present a physical scenary for corroborating the broadband photoresponse of PG materials, as **Figure** **1** depicts. When the photon energy of the laser is much larger than the PG, the photoexcited electrons have much higher energy than the conduction band edge, therefore lose most of their energy by electron‐electron and electron‐phonon scattering before they reach the conduction band edge and contribute to the photocurrent. Such high ratio of energy loss leads to a low photocurrent generation efficiency. As the photon energy approaches the size of PG, the energy loss from the scattering is almost suppressed, causing a high photocurrent generation efficiency. While the photon energy is smaller than the PG, since there is still enough DOS within the gap for photoexcitation, the photocurrent generation efficiency would not be decayed heavily, which is in sharp contrast to the real semiconductor gap. Under this picture, the photoresponsivity for a PG material exhibits uniformly high values across the whole wavelength spectrum, which is the key finding of our work.

Recently, (TaSe_4_)_2_I nanowire was reported with high responsivity of 0.792 A W^−1^ at near‐infrared region,^[^ ^23^ ^]^ but the research including photoresponse mechanism was still at an infant stage. Additionally, low‐noise current level characteristics at dark state were observed in (TaSe_4_)_2_I nanoribbons recently,^[^ ^24^ ^]^ which would be benefit for obtaining high detectivity in photodetection application. Therefore, it is desirable to explore optoelectronic properties further in the long wavelength range.

In this work, we have prepared the (TaSe_4_)_2_I nanoribbons by mechanical exfoliation method, the minimum thickness and maximum length can reach up to 6 nm and 312 µm respectively. We investigated the photoresponse of (TaSe_4_)_2_I over broadband region. Different from the traditional narrow bandgap semiconductor, the increased spectral weight under photoexcitation can be extended within the PG, which leads to high responsivity from λ = 375 nm to λ = 10.6 µm derived from the high‐quality exfoliated (TaSe_4_)_2_I nanoribbon devices. Both the major photoconduction and the minor bolometric mechanism contribute to the photoresponse, which is unambiguously clarified by time‐resolved pump‐probe measurements. To the best of our knowledge, our work is the first one to report such broadband superior responsivity (From 23.9 to 8.31 A W^−1^) at MWIR and LWIR region in 1D single nanoribbon system. Our results demonstrate that quasi‐1D PG materials are promising for MWIR and LWIR photodetection at RT.

## 2 Results and Discussion

(TaSe_4_)_2_I has a monoclinic unit cell (space group I422) that consists of TaSe_4_ chains with helical symmetry that are placed in the middle of the faces and separated by chains of iodine atoms,^[^ ^23^, ^24^ ^]^ the crystal structure is shown in Figure S1a (Supporting Information). In this work, high‐quality (TaSe_4_)_2_I single crystal was synthesized by one‐step chemical vapor transport method, a stoichiometric mixture of Ta and Se were used, with an excess of iodine to be served as both reactant and transport agent, as illustrated in Figure S1b (Supporting Information). The bottom figure of Figure S1b (Supporting Information) shows the typical optical image of as grown (TaSe_4_)_2_I needle‐like single crystals in the cold zone region. The X‐ray diffraction (XRD) spectrum as shown in Figure S1c (Supporting Information) confirms the pure phase composition and high crystal quality. The energy‐dispersive X‐ray spectroscopy reveals a Ta:Se:I ratio of 1.9:7.4:1 (Figure S2, Supporting Information). Due to the weak interchain binding energy in (TaSe_4_)_2_I, it is very easy to obtain the (TaSe_4_)_2_I nanoribbon by using traditional mechanical exfoliation (MF) method. Figure S3a (Supporting Information) shows the typical optical image of exfoliated (TaSe_4_)_2_I nanoribbon/nanoplate, which the thickness was confirmed by AFM. The minimum thickness of nanoribbon we obtained was ≈6 nm (Figure S3b, Supporting Information), and the longest nanoribbon can reach submillimeter level (≈312 µm‐long, 22 nm‐thick), as shown in Figure S3c (Supporting Information), which surpasses most reported thin nanoribbon (below 50 µm‐long) preparedby MF,^[^ ^23^, ^24^, ^25^ ^]^ liquid phase exfoliation (LPE)^[^ ^26^ ^]^ or chemical vapor deposition (CVD) method.^[^ ^5^, ^27^ ^]^

The smooth surface of exfoliated (TaSe_4_)_2_I nanoribbon with different width was confirmed by the scanning electron microscopy (SEM) image, as shown in Figure S4a (Supporting Information). The high‐resolution transmission electron microscopy (HRTEM) of exfoliated thin (TaSe_4_)_2_I shown in Figure S4b (Supporting Information) indicates an interplanar lattice spacing of 7.0Å, and the selected area electron diffraction (SAED) pattern (insert of Figure S4b, Supporting Information) with sharp diffraction spots confirms the high‐quality single‐crystalline structure of (TaSe_4_)_2_I. The Raman spectrum taken on a freshly exfoliated (TaSe_4_)_2_I single crystal is shown in Figure S4c (Supporting Information), which confirms no sample degradation after thinning.

**Figure 2:**
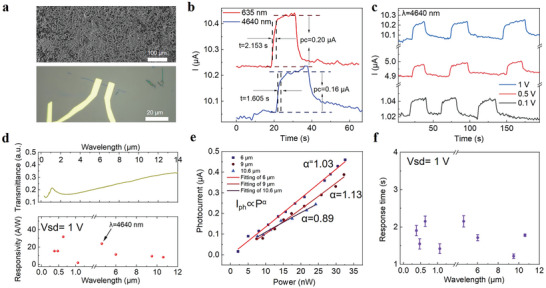
Photo response characterization of (TaSe_4_)_2_I. a) SEM image of the prepared sample on CaF_2_ substrate (top panel) and optical image of the prepared exfoliated nanoribbon device on 285 nm SiO_2_/Si substrate (down panel, sample S1, Supporting Information). The channel area is ≈1um^2^. b) Typical photoresponse under λ = 635 nm and *λ* = 4.64 µm excitation based on (TaSe_4_)_2_I nanoribbon device. c) Voltage‐dependent photoresponse under λ = 4.64 µm excitation. d) Top panel: Transmittance spectra from UV to LWIR region. Down panel: Photo responsivity from UV to LWIR region. e) Power‐dependent photo response in LWIR region. f) Photo response time from UV to LWIR region. The power density is fixed ≈6.7 mW mm^−2^ for (b–d) and (f).

Next, to investigate the potential optoelectronic applications of (TaSe_4_)_2_I, as **Figure** **2**a depicts, large area LPE samples for absorption measurement and two‐probe configuration photodetectors based on freshly MF (TaSe_4_)_2_I nanoribbon were fabricated respectively. We studied power‐dependent and wavelength‐dependent photoresponse of (TaSe_4_)_2_I nanoribbon device over a wide wavelength range at RT. We have completed all photoresponse measurements of (TaSe_4_)_2_I nanoribbon under a bias voltage of 1 V, and the laser polarization is parallel to the chains unless it is specially mentioned. Figure 2b shows the photoresponse under λ = 635 nm and λ = 4640 nm of laser excitation respectively, with the laser power intensity keeping at the same value of 6.7 mW mm^−2^. Surprisingly, we find the photocurrent is only slightly attenuated from visible to MWIR region. The phenomenon is in stark contrast to the traditional semiconductor, which the photocurrent decreases quickly when the photon energy approaches the bandgap. High photoresponsivity (*R*) of 23.9 A W^−1^ was obtained under λ = 4640 nm excitation, which defined as *R* = *I*_pc_/*P* (*I*_pc_ refers to photocurrent and *P* is the power irradiation on the device channel). In addition, bias‐dependent photocurrent is investigated as shown in Figure 2c, the results indicate that the photoresponsivity can be optimized by increasing bias voltage. The obtained photoresponsivity under UV to LWIR range radiation are summarized in Figure 2d (down panel). The variation trend of responsivity almost fits well with the absorption derived from the transmittance spectrum as shown in top panel of Figure 2d. The photoresponse in LWIR region is shown in Figure S5 (Supporting Information). By fitting the photocurrent with an increasing power‐law plot using single power exponent function (*I*_ph_ = *mP^α^*), the almost linear power‐dependent photocurrent is observed in the nanoribbon device (Figure 2e), indicating the photoconductive mechanism dominates.^[^ ^6b^ ^]^ Other mechanism for slow response time such as bolometric effect can be excluded for the main reason in (TaSe_4_)_2_I nanoribbon. Bolometric effect induced slow thermal transport plays a secondary role due to the fast heat dissipation in thin nanoribbon, compared with wide‐thick nanoplate sample (Figure S7, Supporting Information). Also, the measured R‐T curve of (TaSe_4_)_2_I nanoribbon shows a small TCR (≈1.22%/K), indicating the small contribution from the bolometric effect (Figure S6, Supporting Information). The response time, that is, rise time versus wavelength was summarized in Figure 2f. The relative slow response time is attributed to the defect‐induced trap states in nanoribbon,^[^ ^28^ ^]^ which we analyzed its origin in detail in supplementary Note S1 (Supporting Information).

**Figure 3:**
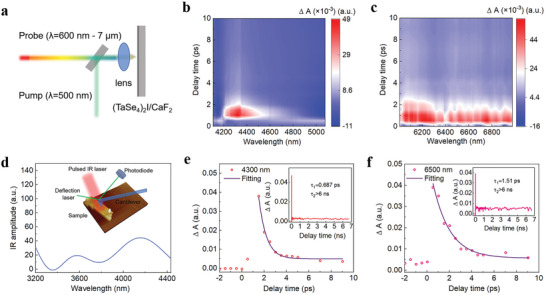
The photo response mechanism of (TaSe_4_)_2_I nanoribbons. a) TA setup. b) 2D TA spectrum under probe wavelength range of 4100–5100 nm. c) 2D TA spectrum under probe wavelength range of 6000–7000 nm. d) Nano‐FTIR absorption measurement under MWIR pulsed laser excitation on a 80 nm‐thick sample. e,f) Typical extracted carrier dynamics at probe wavelength of λ = 4300 and 6500 nm.

To reveal the photoresponse mechanism more clearly, we performed the broadband time‐resolved transient absorption (TA) spectrum measurements. The TA technique can direct probe the broadband carrier relaxation process, which is in contrast with the indirect evidences derived from power‐dependent photocurrent or temperature‐dependent electric transport measurement. The TA setup is illustrated in **Figure** **3**a, the excitation wavelength is λ = 500 nm produced by fundamental beam pumping optical parametric amplification (OPA). The probe wavelength from visible to MWIR region (600 nm–7 µm) is provided from a supercontinuum laser beam and another OPA. The obtained 2D TA spectrum under probe wavelength of 600–900 nm, 1200–1600 nm, 4–5 µm and 6–7 µm are presented in Figure 3b,c and Figure S9a,b (Supporting Information) respectively. Interestingly, different from the negative TA signal (∆A) within near‐infrared region, the TA signal in Figure 3 is positive, indicating a photoinduced absorption phenomenon occurs in MWIR and LWIR region. Combining with analysing the measured broadband transmittance results (Insert of Figure 2d), we confirm the existence of PG, in accordance with the value of≈300 meV at RT derived by previous tr‐ARPES measurement.^[^ ^20^, ^22^ ^]^ Furthermore, a strong TA signal ≈4.3 µm was observed (Figure 3b), indicating a large photoconductivity, which is possibly associated with photo‐excited single polaron states within PG.^[^ ^29^ ^]^ The nano‐FTIR measurement on a 80 nm‐thick sample under pulsed laser excitation also reveals a peaked photo absorption ≈4.3 µm (Figure 3d). The extracted carrier dynamics by typically selected probe wavelength of 4.3 and 6.5 µm are shown in Figure 3e,f respectively. The TA kinetics show a typical characteristic of fast and slow decay time. The dominated fast region contributed to the ultrafast large photoconductivity, the slow part (beyond 6 ns) resulted from the accompanied heat by elevated lattice temperature, due to the sample‘s poor thermal conductivity. The carrier relaxation time under probe wavelength of λ= 4.3 µm displays a decay time of 687 fs by single‐exponential exponent fitting, agreeing with the tr‐ARPES measurement reported recently.^[^ ^20^ ^]^ By increasing probe wavelength toward LWIR region, the fast decay TA signal prolongs, that is doubled to 1.51 ps at λ=6500 nm (Figure 3f), which suggests a slowing down of the photocarriers transportation. Together with the reduced subgap absorption, the photoconductivity becomes suppressed at the LWIR region, leading to decayed photoresponsivity, which is about half of that at 4.3 µm. Nevertheless, the photoresponsivity maintained to LWIR is still high compared to that of other low dimensional materials. The reason why such photoresponsivity is not suppressed sharply to zero when photon energy is below the PG size calls for further investigation, probably due to the finite DOS excitation of PG nature instead of the zero DOS state in conventional semiconductors.

**Figure 4:**
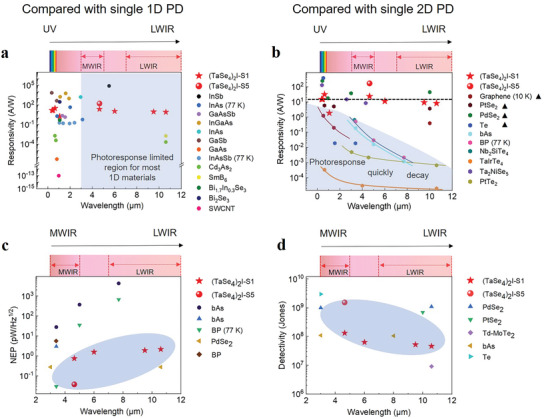
Responsivity, NEP, and detectivity comparison with reported single low‐dimensional materials‐based photodetectors.^[^ ^4^, ^5^, ^6^, ^27^, ^30^, ^31^, ^37^ ^]^ a) 1D PD is short for quasi‐1D 1D nanowire/nanoribbon‐based photodetectors; b) 2D PD is short for 2D material‐based photodetectors). c) NEP value comparison based on low‐dimensional materials worked within MWIR and LWIR region.^[^ ^4^, ^37^, ^38^ ^]^ d) Measured detectivity comparison with reported single 2D material‐based photodetectors from MWIR region to LWIR region.^[^ ^6^, ^37^, ^38^, ^39^ ^]^ The working temperature is room temperature unless it is specially labeled. The lines in Figure 4b are guiding to the eyes. Symbol ▲ indicates the defect related trap states to tune the photoresponse.

Overall, the (TaSe_4_)_2_I nanoribbons demonstrate high photoresponsivity especially in MWIR and LWIR region. For example, the responsivity of ≈23.9 A W^−1^ @ 4.64 µm is two orders of magnitude higher than that of other 2D Weyl semimetal TaIrTe_4_,^[^ ^30^ ^]^ 3D Weyl semimetal TaAs^[^ ^5a^ ^]^ and 1D narrow‐bandgap semiconductor InAsSb^[^ ^31^ ^]^ operating at 77K. The responsivity could be further improved by optimizing the sample geometry, i.e. ≈170 A W^−1^@ 4.64 µm obtained in another nanoribbon device (sample S5, Figure S10, Supporting Information). The photoresponsivity of our nine fabricated devices are shown in Figure S11 (Supporting Information). The photoresponsivity also outperforms the commercialized HgCdTe detector (Responsivity of 0.2–1.7 A W^−1^) worked at liquid nitrogen temperature,^[^ ^1a^ ^]^ far beyond most reported low‐dimensional based MWIR photodetectors. A more detailed photoresponsivity comparison with broadband range based on single low‐dimensional materials is summarized in **Figure** **4**. Only intrinsic photoresponsivity of single material is considered here (without any treatment like applying plasmonic structure, ferroelectric polymers or composed as a heterostructure). To the best of our knowledge, for single 1D systems, our work is the first one to report such ultra‐broadband high responsivity with wavelength range covering λ = 375 nm to λ = 10.6 µm, especially with record‐high values in LWIR region at RT. Even compared with 2D materials, our device also outperforms most of them from MWIR to LWIR region.

According to Fermi golden rule, the photo‐excited interband transition is related to DOS and transition matrix element, to be more exactly, the absorption α∝*g*(*hv*)|*M_cv_* |^2^,^[^ ^32^ ^]^ where *g*(*hv*) is the joint DOS involving both conduction and valence bands, and *M_cv_* is the transition matrix element. The DOS for a thick material displays a square‐root decaying behavior towards the bandgap edge,^[^ ^33^ ^]^ thus the absorption coefficient of most semiconducting film materials quickly decays. While for (TaSe_4_)_2_I, the DOS does not decay much since the pseudogap nature of the room temperature ground state. Interestingly, (TaSe_4_)_2_I is also claimed to be a Weyl semimetal at room temperature,^[^ ^34^ ^]^ thus can also keep a slow absorption decaying, due to the linear dispersion of the band structure in low energy region, which is similar to the case of Dirac semimetal graphene,^[^ ^35^ ^]^ by considering the joint DOS and transition matrix element.

The photoresponsivity (*R*) for photodetector based on low‐dimensional material can be expressed as^[^ ^36^ ^]^:

(1) $$R=\frac{I_{pc}}{P}=\frac{et\alpha }{hv}\cdot \frac{\tau_{l}}{\tau_{t}}$$

where *e* is the elementary electron, *t* is the sample thickness, *α* is the absorption coefficient, *h* is Planck's constant, *v* is light frequency, *τ*_l_ is carrier lifetime, *τ*_t_ is the electron transit time. For (TaSe_4_)_2_I, the coefficient$\frac{\alpha }{hv}$ is not varied much as the photon energy decreases when the light polarization is parallel to the |TaSe_4_| chains.^[^ ^29^ ^]^ Owning such relatively high absorption in long wavelength region enables (TaSe_4_)_2_I to possess equivalently high photoresponsivity in ultra‐broadband region, in contrast to the fast decaying behavior near the bandgap edge for most thick 2D semiconductor materials, as displayed in Figure 4b.

Futhermore, the Fourier transform infrared spectrum (FTIR) spectrum (Insert of Figure 2d) demonstrates the optical absorption of (TaSe_4_)_2_I beyond 10 µm, indicating a great potential for realizing high responsivity in far‐infrared band.

The noise equivalent power (NEP) is another important figure of merit to evaluate the weak light detection performance. The lower NEP, the photodetector is more sensitive. It can be expressed as *NEP* = *RMS*(*I*_noise_)/*R*, where *I_noise_* is the noise current of the device, *RMS* is the root mean square. The measured noise current spectrum is shown in Figure S12 (Supporting Information). The typical NEP value obtained is ≈753 fW/Hz^1/2^@ 4.64 µm (sample S1, Supporting Information), while the highest parameter we can achieve is ≈38 fW/Hz^1/2^@ 4.64 µm (sample S5, Supporting Information), which is almost the lowest value among single low‐dimensional materials within MWIR region at RT, as shown in Figure 4c. The detailed detectivity comparison within MWIR and LWIR range is summarized in Figure 4d (2D) and Figure S13 (Supporting Information) (1D), which is also competitive. In addition, the availability of blackbody response in an infrared photodetector is critical for practical applications. The detail about the blackbody response test of (TaSe_4_)_2_I nanoribbon is shown in supplementary note 3. Under a bias voltage of 0.1 V, the responsivity of the device is obtained ≈17 A W^−1^ under 1200 K blackbody source illumination, which is superior than most low‐dimensional blackbody‐sensitive photodetectors.^[^ ^40^ ^]^ The measured detectivity is ≈1.56 × 10^8^ Jones, which is higher than that of 1D carbon tube and comparable with 2D Te.^[^ ^40b,c^ ^]^ The detectivity can be further improved by specially designing the heterostructure with other 2D materials.^[^ ^41^ ^]^ Though the response time is relatively slow at present stage, on one hand, there's often a trade‐off between responsivity and response time, the defects of the sample could be reduced by further iodization treatment through annealing in iodine atmosphere.^[^ ^42^ ^]^ On the other hand, beyond photodetection application, the fall time with a relative long tail ≈20 s was observed due to the charge de‐trapping process, the persistent photoconductivity (PPC) phenomenon could be exploited for novel optoelectronic synapses and optical memory application.^[^ ^43^ ^]^ Although not all the parameters bear the best values, the proposed new type of broadband photodetector based on quasi‐1D PG system, may provide a new route to achieve uncooled high‐performance broadband photodetector.

## 3 Conclusion

In summary, we demonstrated a new type of ultrabroadband high photoresponsivity photodetector based on PG system (TaSe_4_)_2_I. Due to the increased spectral weight under photoexcitation extended to PG region, ultrabroadband high photoresponse from 375 to 10.6 µm was demonstrated based on single (TaSe_4_)_2_I nanoribbon. Furthermore, the broadband photoexcited carrier dynamics were revealed in our TA experiments, demonstrating that the contribution to the high photoresponsivity is from the absorption of single polaron states within the PG and majorly photoconductive mechanism. The typical nanoribbon device shows high photoresponsivity especially in MWIR and LWIR regions (from 23.9 to 8.31 A W^−1^ with a bias voltage of 1 V) at RT. The best performance (photoresponsivity of 170 A W^−1^, NEP of 38 fW//Hz^1/2^ and detectivity of 1.54 × 10^9^ Jones) we achieve is very competitive among single low‐dimensional material based MIWR photodetectors. In addition, it is also demonstrated to have a large blackbody response. Such balanced performance in broadband IR region enables (TaSe_4_)_2_I to be a potential candidate material for high performance broadband IR photodetector, like QCD. Our work thus paves a way for exploring low manufacturing cost, high‐performance MWIR and LWIR photodetector at RT by using quasi‐1D PG materials.

## 4 Experimental Section

### Materials Synthesis

High‐quality single crystals of (TaSe_4_)_2_I were synthesized by chemical vapor transport (CVT) method in a sealed quartz tube. The high‐purity Ta(4N), Se(4N), and I(4N) were mixed in chemical stoichiometry sealed in an evacuated quartz tube which inserted into a furnace with a temperature gradient of 500 to 400 °C with the educts in the hot zone. After 2 weeks, shiny crystals with needle‐like shape were obtained in the cold zone. The thin (TaSe_4_)_2_I nanosheets synthesized by liquid phase exfoliation (LPE) method were prepared for broadband absorption measurement and directed deposited on a Cu grid for TEM characterization.

### Materials Characterization

HRTEM analysis was carried out on a JEM2100F with an acceleration voltage of 200 kV. The SEM and EDX characterizations were performed in an Oxford SEM system. Raman spectroscopy was performed on a freshly cleaved (TaSe_4_)_2_I under a 100× objective lens by using a grating of 1800 g mm^−1^. To avoid the laser‐induced damage of the samples, the optimized Raman spectrum were recorded at low power level (P ∼ 500 µW). For broadband optical absorption analysis, the transmittance spectra were measured by a UV‐NIR spectrometer (Agilent Cray 5000) and a FITR (Vertex 70) spectrometer under at room temperature. The nano‐FTIR spectrum were measured from a multi‐functional nano‐infrared spectrometer (Anasys Instruments Inc.).

### Device Fabrication

For fabrication of the nano‐thick devices, electrode patterns were defined by standard electron beam lithography. Metal electrodes (10 nm Cr/100 nm Au) were deposited by thermal evaporation in PVD system (K.J.Lesker Nano 36). The thickness of (TaSe_4_)_2_I nanoribbons were determined by atomic force microscopy under non‐contact mode (Park NX‐10).

### Electrical and Photo Response Measurement

The current–voltage (*I*–*V*) measurements were performed under voltage driving mode along the chain direction. The photoelectric signal and photo response time under biased voltage were measured by using a Keithley 2450 sourcemeter. For wavelength‐dependent photocurrent measurements, different continuous‐wave solid‐state lasers (Changchun New Industries Optoelectronics Technology Ltd. λ = 375 nm, 437 nm, 635 nm, 1064 nm), a single‐wavelength (λ = 4.64 µm) and a wavelength‐tunable (λ = 6‐10.6 µm) mid‐IR continuum wave quantum cascade laser (Daylight Solutions) were used as light sources. The incident light power illuminated on the device was monitored by calibrated power meters. The laser spot diameters were ≈3, 4, 4, 1, 3.9, and 2 mm for 375 nm, 437 nm, 635 nm, 1064 nm, 4.64 µm, and 6–10.6 µm, respectively. A 1200 K blackbody source (HGH RCN1250) was used to detect blackbody response of the device.

## Conflict of Interest

The authors declare no conflict of interest.

## Supporting information

Supporting Information

## Data Availability

The data that support the findings of this study are available from the corresponding author upon reasonable request.
