# Supplementary material for: Ultrabroadband High Photoresponsivity at Room Temperature Based on Quasi‐1D Pseudogap System (TaSe4)2I
Source: Adv Sci (Weinh). 2023 Dec 8;11(7):2302886. doi: 10.1002/advs.202302886 (PMC10870056; doi:10.1002/advs.202302886)
Supplement: Supplementary file 1 — Supporting Information [file ADVS-11-2302886-s001.pdf]

ADVANCED SCIENCE
Open Access

Supporting Information

for *Adv. Sci.*, DOI 10.1002/advs.202302886

Ultrabroadband High Photoresponsivity at Room Temperature Based on Quasi-1D Pseudogap System $(TaSe_4)_2I$

Jialin Li, Qing Li, Junjian Mi, Zhuan Xu, Yu Xie, Wei Tang, Huanfeng Zhu, Linjun Li and Limin Tong*

Supporting Information

Ultrabroadband High Photoresponsivity at Room Temperature Based on Quasi-1D Pseudogap System $(TaSe_4)_2I$

Jialin Li, Qing Li, Junjian Mi, Zhuan Xu, Yu Xie, Wei Tang, Huanfeng Zhu, Linjun Li, Limin Tong*

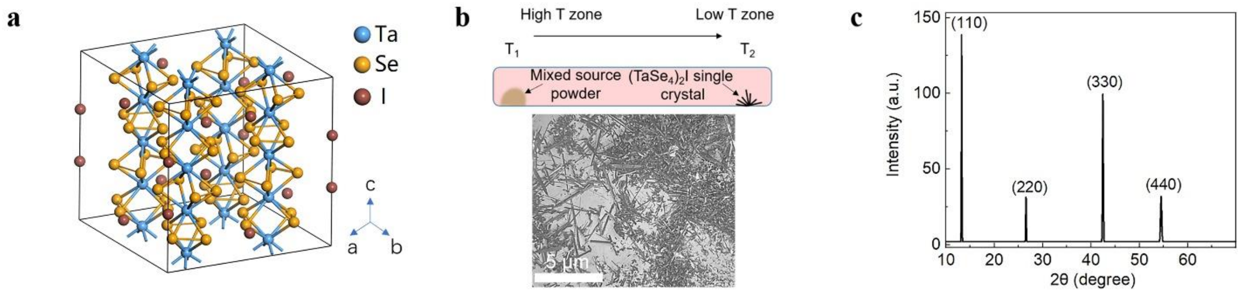

Figure S1. Crystal structure, growth and XRD characterization of $(TaSe_4)_2I$. (a), Crystal structure of $(TaSe_4)_2I$. (b), Growth method and optical image of as grown $(TaSe_4)_2I$ single crystal. (c), XRD spectrum of $(TaSe_4)_2I$ single crystal.

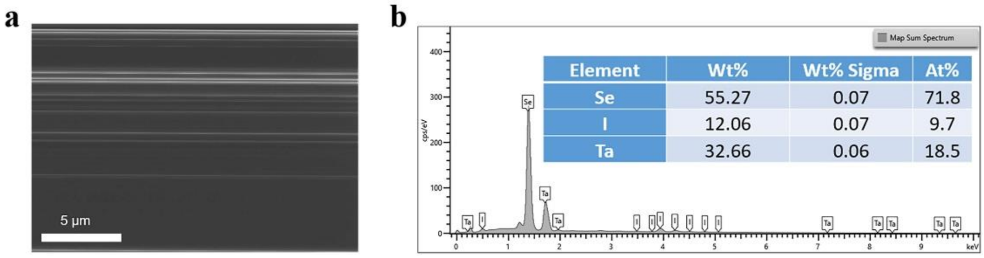

Figure S2. SEM and EDX characterization of $(TaSe_4)_2I$ bulk single crystal. (a), SEM image. (b), EDX analysis.

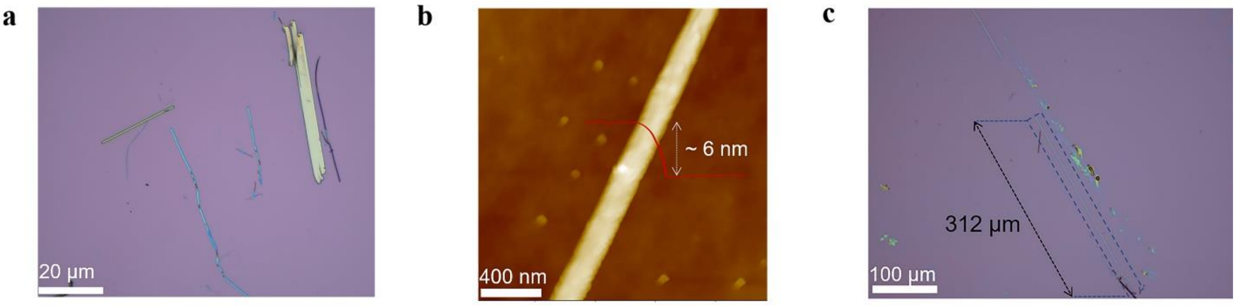

Figure S3. Typical images of mechanic exfoliated $(TaSe_4)_2I$ nanribbon/nanoplate. (a), Optical image of typical mechanic exfoliated $(TaSe_4)_2I$ nanoribbon/nanoplate. (b), AFM image

of the obtained thinnest nanoribbon sample. (c), Optical image of the obtained longest nanoribbon sample.

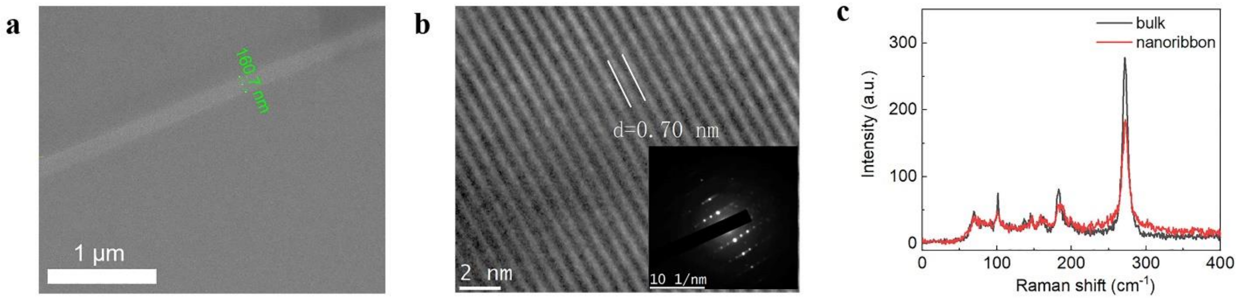

Figure S4. SEM, HRTEM and Raman characterization of exfoliated $(TaSe_4)_2I$ nanoribbon. (a), SEM image of typical mechanic exfoliated $(TaSe_4)_2I$ nanoribbon. (b), HRTEM image of the nanoribbon sample. Insert figure is SAED of the nanoribbon sample. (C), Raman spectrum of bulk and thin nanoribbon (~ 32 nm-thick) sample.

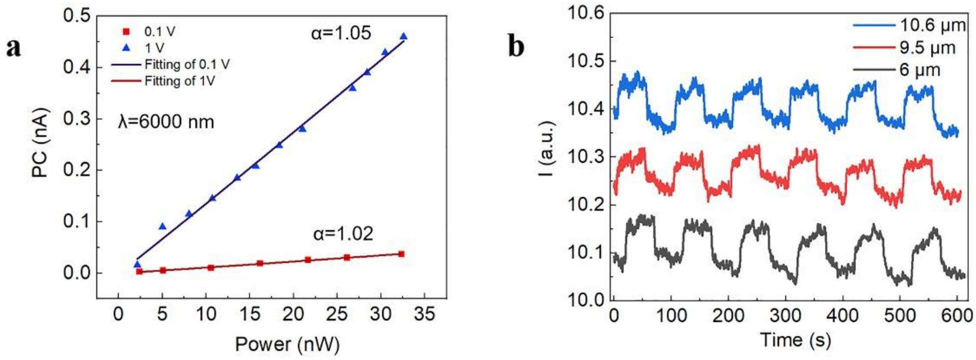

Figure S5. Photo response beyond MWIR region. (a), Voltage-dependent photocurrent (PC). (b), Light on/off cycles under power density of 6.7 mW/mm^2 with bias voltage of 1V.

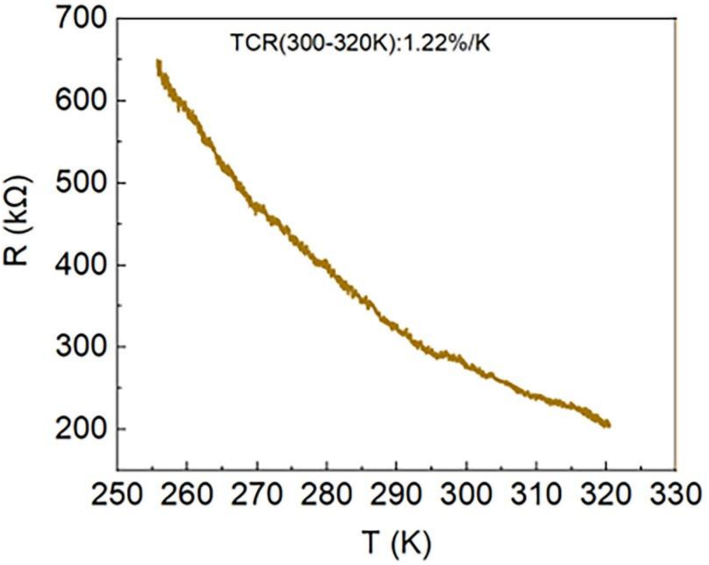

Figure S6. R-T curve of a nanoribbon device above T_{cdw}.

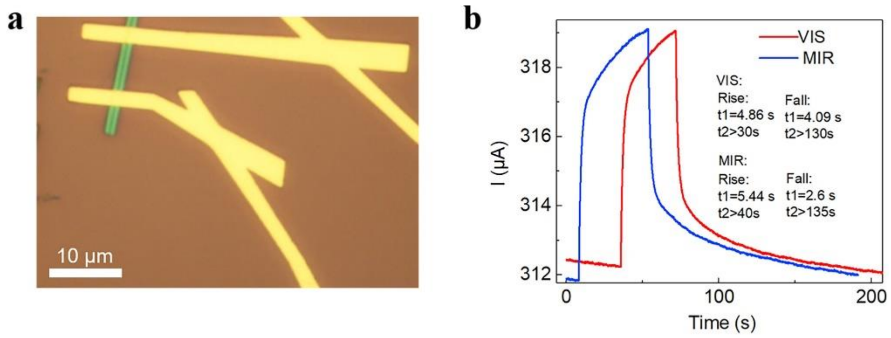

Figure S7. Photo response of an exfoliated $(TaSe_4)_2I$ nanoplate device. (a), Optical image of typical mechanic exfoliated $(TaSe_4)_2I$ nanoplate. (b), Photo response of the nanoplate sample under visible (VIS) light (λ=635 nm) and mid-infrared (MIR) light (λ=4600 nm).

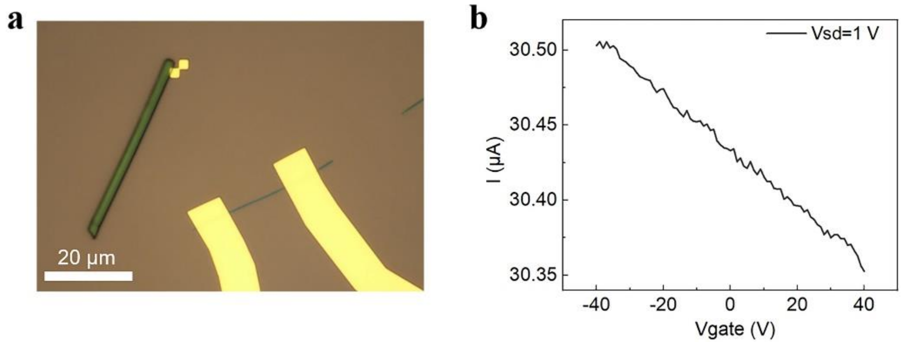

Figure S8. Typical transfer curve of an exfoliated $(TaSe_4)_2I$ nanoribbon device. (a), Optical image. (b), Transfer curve at dark state.

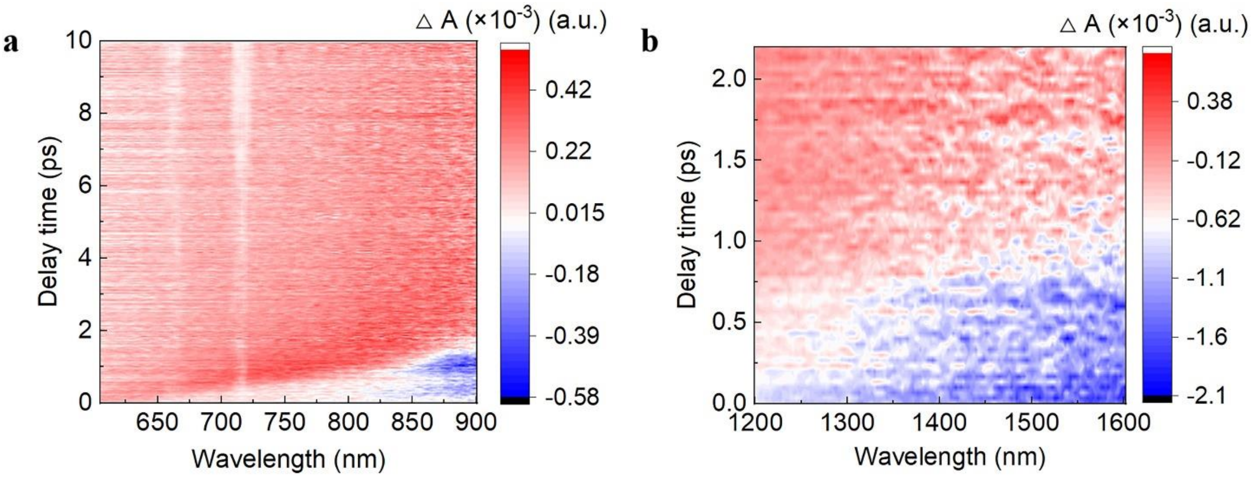

Figure S9. 2D TA spectrum at probe wavelength of visible and near-infrared region under λ=500 nm pump. (a), Visible region. (b), Near-infrared region.

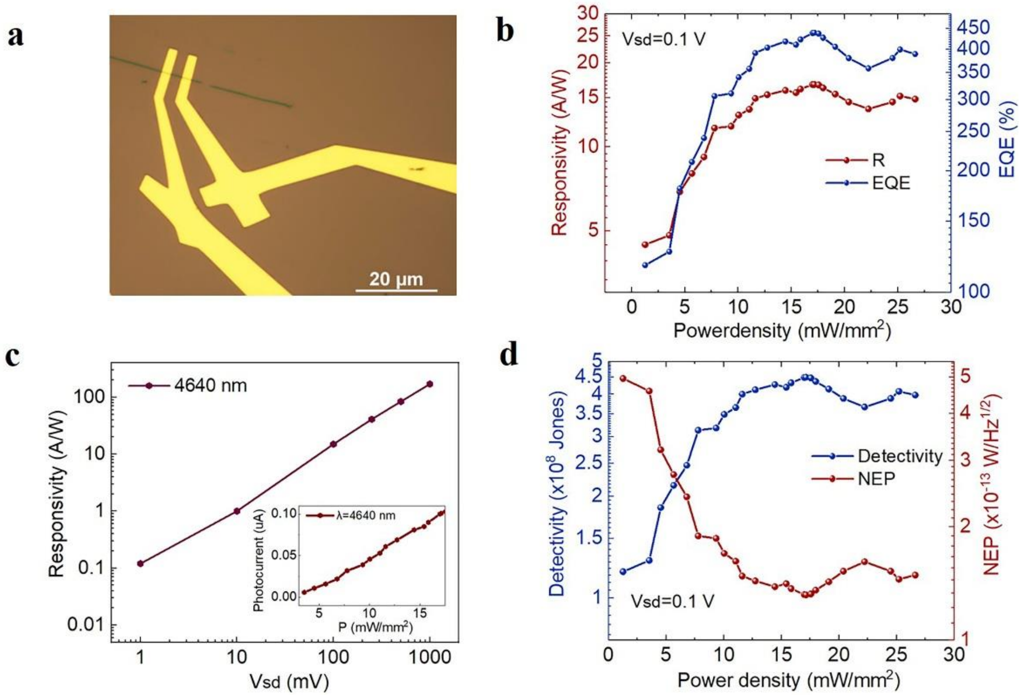

Figure S10. Photoresponse of an optimized sample (Sample S5). (a), Optical image. (b), Photo responsivity and EQE obtained under λ = 4640 nm laser excitation with bias voltage of 0.1 V. (c) Voltage-dependent photoresponsivity under λ = 4640 nm laser excitation. Insert: power-dependent photocurrent. (d) Detectivity and NEP obtained under λ = 4640 nm laser excitation with bias voltage of 0.1 V.

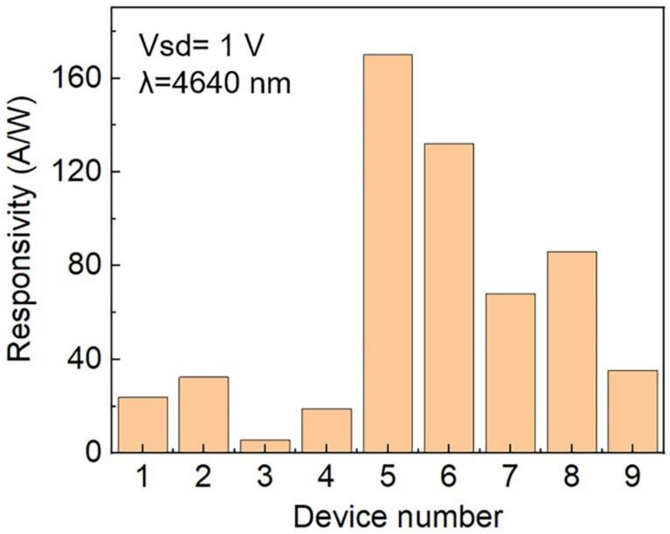

Figure S11. Responsivity obtained at λ=4.64 μm in nine $(TaSe_4)_2I$ nanoribbon device with different geometry under bias voltage of 1V at RT.

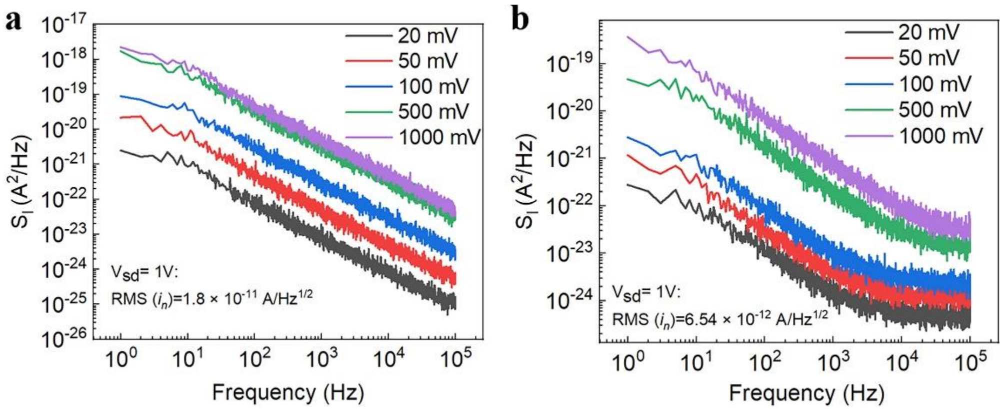

Figure S12. Noise current spectrum of $(TaSe_4)_2I$ nanoribbon devices under different bias voltage. (a), Unoptimized device (Device S1 in Figure2). (b), Optimized device (Device S5 in FigureS8).

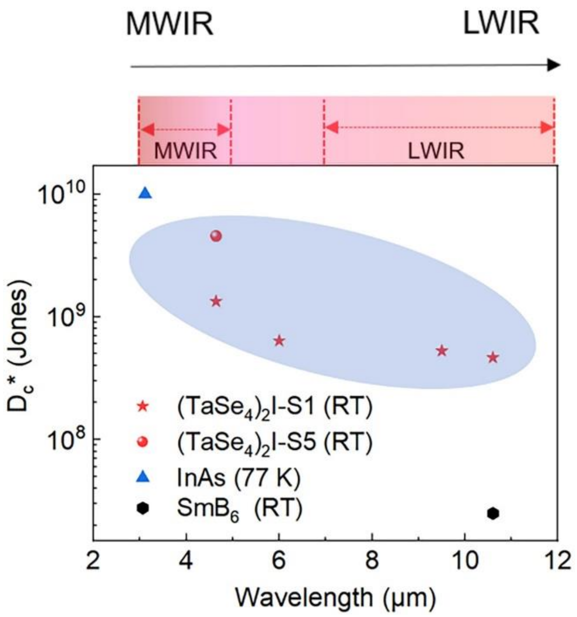

Figure S13. Calculated detectivity D_c* comparison with reported single 1D material-based photodetectors from MWIR region to LWIR region[1]. More calculation process description is shown in supplement note.

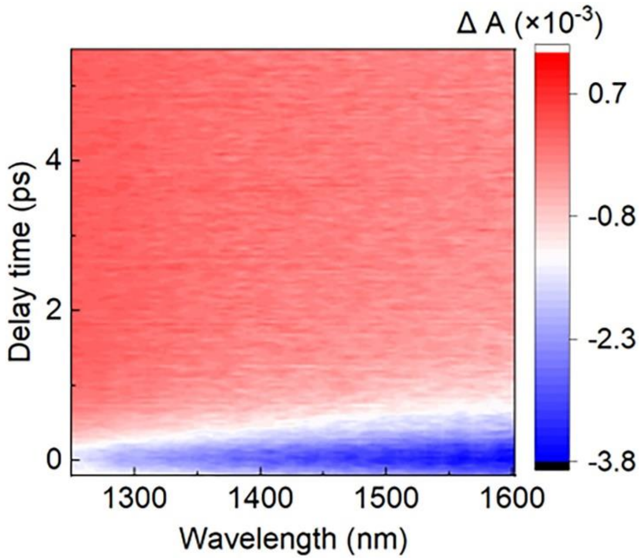

Figure S14. 2D TA spectrum at probe wavelength of near-infrared region under λ=1030 nm pump. The dynamics is almost the same as that under λ=500 nm pump, indicating the laser pump energy does not affect much about the carrier relaxation process.

Supplementary Note 1

Origin of defects in $(TaSe_4)_2I$:

The trap state in $(TaSe_4)_2I$ is majorly originated from the three aspects:

1. The defects from the single crystal growth and exfoliation process. From the crystal structure view as shown in Fig.S15, there is an isolated unbonded iodine ion as the black arrows points to. Therefore, it probably introduces the iodine vacancy during the crystal growth period and exfoliation process.

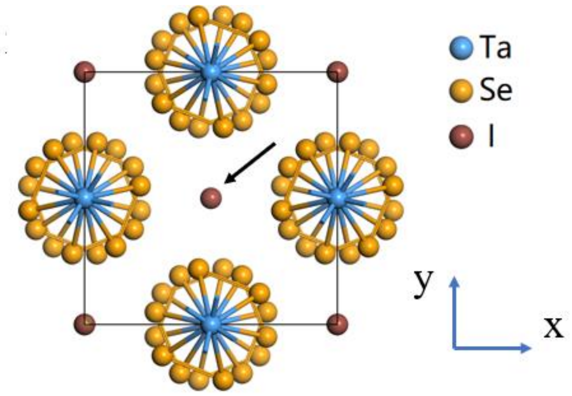

Figure S15. Crystal structure of $(TaSe_4)_2$.

2. The unbonded iodine ion movement under bias voltage. As illustrated in Fig.S16a, the unbonded iodine ion under bias voltage are easy to move, and the movement process will destroy the lattice of the material, introducing more point defects (Iodine vacancy/iodine gap). To observe the iodine ion movement more obviously, we conducted an experiment under high electric field of 2×10^6 V/m, the I-T curve shows that the dark current decreases with time (The device is protected by PMMA, which is isolated from air), as shown in Fig.S16b, indicating the unbonded iodine ion moves as the time increases, similar as the phenomenon of perovskite materials containing iodine working in bias voltage mode[2].

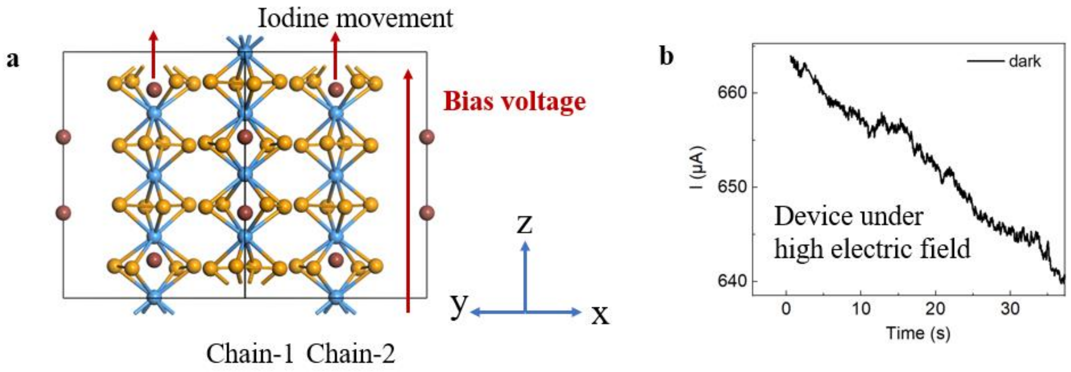

Figure S16. Iodine ion movement of $(TaSe_4)_2$ under biased voltage.

3. The photo excitation process. Under photo excitation, the photogenerated carriers will further cause iodine atoms to move, which will further introduce more defects and reduce the mobility of materials. Therefore, when the light is removed, the detrapping time of the photogenerated carriers is much slower.

Supplementary Note 2

Detectivity calculation:

Detectivity is a figure of merit used to characterize the device ability to respond to weak incident signals, given as $D^* = \sqrt{AB}/NEP$, where *NEP* is the noise equivalent power, *A* is the channel area and *B* is the bandwidth. The *NEP* is related with the noise current, which can be expressed as $NEP = I_{\mathrm{noise}}/R$. Here we define the detectivity extracted from this method to be the measured detectivity (D_M*). For example, the I_{noise} of the device obtained from Figure S12a is about 1.8×10^{-11} A/Hz$^{1/2}$, the photoresponsivity of the device in Figure 2 is 23.9 A/W, thus the NEP value is about 753 fW//Hz$^{1/2}$@ 4.64 μm. Therefore, the measured detectivity D_M* is 1.33×10^8 Jones@ 4.64 μm. Another device (S5) calculated is about 1.54×10^9 Jones@ 4.64 μm by using the similar method.

But in the research field of low-dimensional materials-based photodetectors, some works don’t measure the noise current spectrum, they consider that the shot noise from dark current is

the major factor limiting detectivity, thus the noise current can be replaced by $I_{noise}=\sqrt{2eI_{dark}}$ at 1 Hz bandwidth, where *e* is the elementary charge and I_{dark} is the dark current, thus the specific detectivity can be simply expressed as $D^{*}=R\sqrt{A}/\sqrt{2eI_{dark}}$.

To make it more convenient to compare the device performances with previous works, the reported detectivity for the same calculation method (D_C*) in 1D system were summarized in FigureS13. There were only few materials reported the detectivity in 1D system within MWIR and LWIR region. The reason behind may be attributed to the limited bandgap or too low photoresponsivity (such as 1D Cd_3As_2 nanowire). As shown in Figure S13, the detectivity is not decayed much from MWIR to LWIR region for $(TaSe_4)_2I$ at room temperature, indicating it has potential trend for high performance photodetection beyond LWIR region. Also, the reported detectivity for the same calculation method (D_M*) in 2D system were summarized in Figure 4d.

Supplementary Note 3

Blackbody response measurement

To check whether $(TaSe_4)_2I$ nanoribbon can be sensitive for blackbody radiation, we did the direct blackbody radiation photocurrent response test. Since blackbody response is often smaller than the response under laser excitation[3], we choose a nanoribbon device with much defects (with high photo gain), to produce relatively large blackbody response. Fig. R17a presents the schematic diagram of the blackbody detection system. Fig. R17b shows optical image of one nanoribbon device. It is prepared by transferring the pre-fabricated gold electrode onto the surface of the $(TaSe_4)_2I$ nanoribbon with PMMA encapsulation. Interdigitated electrodes with 500 nm channel length were used to collect the photo carriers more effectively. Fig. R17c shows the blackbody response of the $(TaSe_4)_2I$ nanoribbon photodetector under 1200 K blackbody source illumination. Under bias voltage of 0.1 V, the responsivity is obtained about 17 A/W under illumination power of 11.8 nW, which is superior than most low-

dimensional blackbody-sensitive photodetectors[4]. The measured detectivity is about 1.56 × 10^8 Jones. The response time is slow due to the defects in $(TaSe_4)_2I$ nanoribbon, which has been detailly discussed in supplementary note 1. By fitting the photocurrent with increasing blackbody power plot using single power exponent function ($I_{ph} = mP^{\alpha}$), the fitting parameter α=0.23 is much smaller than 1, indicating there is much trap states in the device, as shown in Fig. R17d. Since blackbody response bandwidth is also important, further work needs to be investigated for reducing the sample defects.

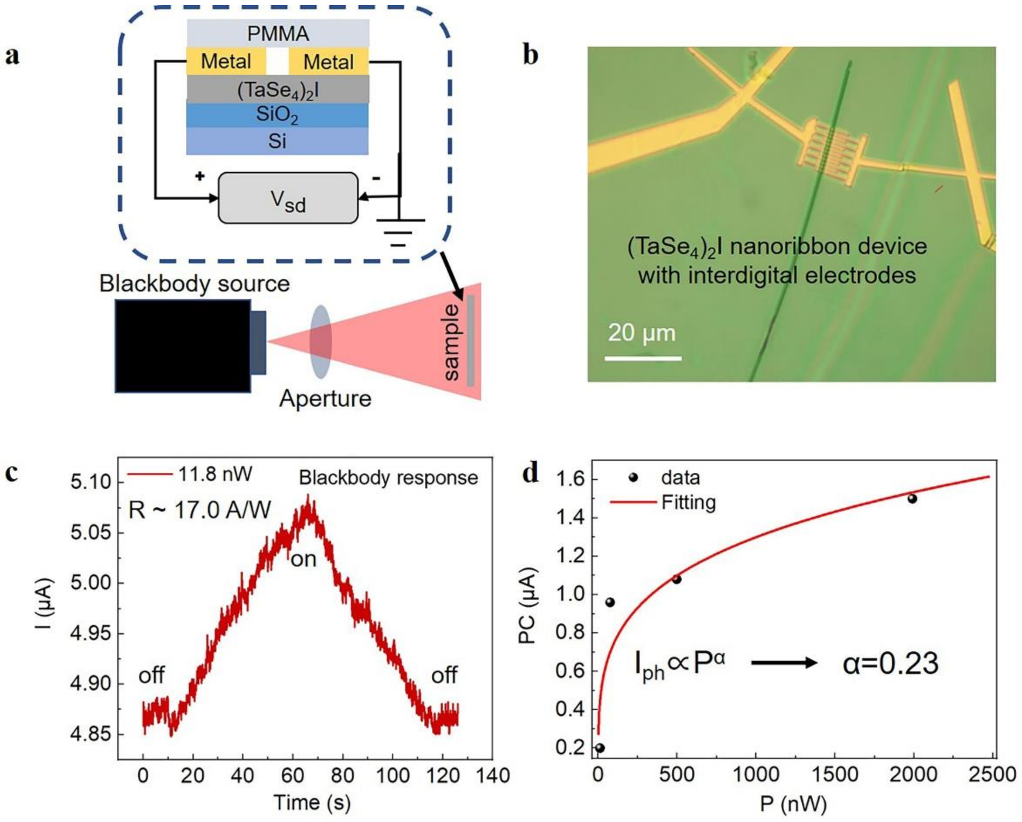

Figure S17. Blackbody response test of $(TaSe_4)_2I$ nanoribbon device

Reference

[1] a)H. Fang, W. Hu, P. Wang, N. Guo, W. Luo, D. Zheng, F. Gong, M. Luo, H. Tian, X. Zhang, C. Luo, X. Wu, P. Chen, L. Liao, A. Pan, X. Chen, W. Lu, *Nano Lett.* **2016**, 16, 6416; b)Y. Zhou, J. Lai, L. Kong, J. Ma, Z. Lin, F. Lin, R. Zhu, J. Xu, S.-M. Huang, D. Tang, S. Liu, Z. Zhang, Z.-M. Liao, D. Sun, D. Yu, *Appl. Phys. Lett.* **2018**, 112, 162106.

[2] Y. Deng, S. Xu, S. Chen, X. Xiao, J. Zhao, J. Huang, *Nat. Energy* **2021**, 6, 633.

[3] F. Wang, T. Zhang, R. Xie, Z. Wang, W. Hu, *Nat Commun* **2023**, 14, 2224.

[4] a)J. Bullock, M. Amani, J. Cho, Y.-Z. Chen, G. H. Ahn, V. Adinolfi, V. R. Shrestha, Y. Gao, K. B. Crozier, Y.-L. Chueh, A. Javey, *Nat. Photonics* **2018**, 12, 601; b)M. Zhang, D. Ban, C. Xu, J. T. W. Yeow, *ACS Nano* **2019**, 13, 13285; c)M. Peng, R. Xie, Z. Wang, P. Wang, F. Wang, H. Ge, Y. Wang, F. Zhong, P. Wu, J. Ye, Q. Li, L. Zhang, X. Ge, Y. Ye, Y. Lei, W. Jiang, Z. Hu, F. Wu, X. Zhou, J. Miao, J. Wang, H. Yan, C. Shan, J. Dai, C. Chen, X. Chen, W. Lu, W. Hu, *Sci Adv* **2021**, 7, eabf7358.
